# Curiosity as a Moderator of the Relationship Between Entrepreneurial Orientation and Perceived Probability of Starting a Business

**DOI:** 10.3389/fpsyg.2022.884092

**Published:** 2022-05-20

**Authors:** Nicolás Pablo Barrientos Oradini, Andrés Rubio, Luis Araya-Castillo, Maria Boada-Cuerva, Mauricio Vallejo-Velez

**Affiliations:** ^1^School of Administration and Business, Miguel de Cervantes University, Santiago, Chile; ^2^Faculty of Economics and Business, Andres Bello University, Santiago, Chile; ^3^Faculty of Psychology, Diego Portales University, Santiago, Chile; ^4^Human Factor, Organizations and Markets (FHOM), Faculty of Business and Economics, Universitat Rovira i Virgili (URV), Tarragona, Spain; ^5^Faculty of Psychology, Universidad de Medellín, Medellín, Colombia

**Keywords:** entrepreneurial orientation, entrepreneurship, curiosity, sense of execution, intrinsic motivation

## Abstract

Although the correlation between Entrepreneurial Orientation (EO) and concrete actions to set up a business or the Probability of Starting a Business (PSB) has been widely studied, the psychological factors that can affect this relationship have not yet been sufficiently addressed in the field of entrepreneurship. One of them is curiosity. Both at theoretical and empirical level, a distinction are usually made between two types of curiosity. I-type curiosity is associated with the anticipated pleasure of discovering something new, and D-type curiosity is associated with reducing uncertainty and eliminating unwanted states of ignorance. Consequently, this paper aims to analyze the moderating role that the types of curiosity play in the relationship between EO and PSB, considering their interaction with sociodemographic variables. The sample of this cross-sectional study consisted of 1,761 participants (convenience sampling of active workers; 49.8% men; mean age 38.88 years, *SD* = 12.53 years; 22.9% Colombian and 77.1% Spanish). The EO scale and a curiosity scale were applied. In addition, participants were asked, based on their perception, how likely they were to start their own business within the next 5 years. A simple moderation analysis was considered to test the moderating role of both types of curiosity in the relationship between EO and PSB. Next, a double moderation analysis was carried out in order to identify, which sociodemographic variables moderate the moderating effect of curiosity. The results show that only the D-type component moderates the relationship between EO and PSB: The higher the D-type curiosity, the stronger the association between EO and PSB. In terms of sociodemographic variables, neither culture nor gender showed a moderating effect on the moderation exerted by D-type curiosity. While, age did moderate the moderating effect of D-type curiosity on the relationship between EO and PSB. Results are discussed in terms of spirituality (attitudes, practices, and behaviors) and the resolution of problems associated with the entrepreneurial process, considering cognitive and psychological factors, particularly with an emphasis on explaining why only D-type curiosity shows a moderating effect. Finally, the limitations of the study and potential future lines of research are pointed out.

## Introduction

In the field of entrepreneurship, interest in the study of factors associated with spirituality (understood as a set of practices and attitudes) and psychological capital (understood as the ability to maintain a positive emotional state in the face of adversity and demands) has been increasing. Likewise, the study of these variables in this field has been acquiring relevance in the context of the COVID-19 Pandemic, considering the importance of psychological factors when explaining individual differences. These factors usually mark the main differences when individuals can see (or not) crisis contexts as opportunity contexts.

Despite the above, there are variables linked to spirituality and psychological capital that have not yet been analyzed in terms of their effects in the context of entrepreneurship. One of these variables is curiosity. For example, the association between Entrepreneurial Orientation (EO) and specific actions to set up a business or the Probability of Setting up a Business (PSB) has been extensively studied (Rauch et al., 2009; Vij and Singh Bedi, 2012; Cho and Lee, 2018), but we do not know how curiosity can affect this relationship.

Curiosity is theoretically understood as the desire of knowledge, which motivates people to learn new ideas, eliminate outdated information, and solve intellectual problems (Loewenstein, 1994). Berlyne (1978), regarded as the first great theorist on curiosity, described it as a human characteristic in the journey toward knowledge that leads to experimentation, intellectual development, and achievement. Curiosity is a dispositional tendency that manifests itself as a personality trait associated with positive emotional-motivational states involving interest and intrinsic pleasure in learning and problem solving. In this regard, curiosity is positively associated with the search for sensations (Hsee and Ruan, 2016; Silvia, 2017), the interest in exploring unfamiliar subjects in order to learn something new (Grossnickle, 2016; Wade and Kidd, 2019), the enjoyment of problem-solving (Kashdan et al., 2018), facing challenges (Geum et al., 2020), and with cognitive activity, as it is a motivator for learning and is crucial in decision-making processes. (Kidd and Hayden, 2015). According to Litman (Litman and Jimerson, 2004; Litman, 2010, 2019; Litman et al., 2010), there are two types of curiosity. The first one is the I-type, which is associated with the anticipated pleasure felt when acquiring new knowledge, simply for the intrinsic pleasure of gaining it. The second one is D-type, which is associated with a wish to lower uncertainty and to eliminate unwanted ignorance. In this second type of curiosity, there is a need to know in which areas the importance of accuracy, precision, and ownership of information is paramount, and it is conceptualized as a state of unfulfilled need. In this type of curiosity, learning is oriented toward performance and solving tangible problems. Considering the above, curiosity seems to be a highly relevant variable when it comes to understanding phenomena linked to motivation, satisfaction, or learning, as in the case of entrepreneurship (Silvia, 2012; Halamish et al., 2019; Murnieks et al., 2020). Although there is already evidence showing the relationship between curiosity and EO (Syed et al., 2020; Boada-Grau et al., 2021), as well as between curiosity and specific entrepreneurial behaviors (Prihatsanti, 2018) there is no evidence on how curiosity can affect the relationship between these two concepts. In this respect, the analysis of how different types of curiosity may moderate the relationship between EO and PSB, i.e., how different levels of curiosity shown by individuals may affect the relationship between EO and PSB is presented in this work as a knowledge gap.

Considering the above, the research problem of this study refers to how the different types of curiosity affect the relationship between EO and PSB. The contribution of this study is to know how a psychological characteristic such as curiosity moderates the relationship between EO and PSB is a key to better understand the entrepreneurship phenomenon. This makes it possible to identify the factors that would make entrepreneurs move from an entrepreneurial orientation to specific entrepreneurial behaviors (such as setting up a company). Furthermore, these types of identified factors (those favoring specific entrepreneurial behaviors) can be used as input for developing pedagogical approaches in the field of entrepreneurship, both in children, adolescents, and adults.

Considering the previous, it is important to understand to what extent these two types of curiosity work as differentiating psychological factors in the process of moving from an entrepreneurial orientation toward more concrete entrepreneurial behaviors. Therefore, this study aims at analyzing the moderating role played by the types of curiosity in the relationship between EO and PSB, considering their interaction with socio-demographic variables, such as age, gender, and culture.

## Materials and Methods

### Participants

This is a quantitative study with a cross-sectional design. The population was defined as adult workers (men and women), with the aim of analyzing the variables in the general population and not only in the population linked to entrepreneurship. The participants were from Spain and Colombia, to analyze possible cultural differences in the relationships between the variables studied. A convenience sample of 1,761 participants of active workers contacted during 2018 and 2019 was considered. The average age of the sample was 38.88 years (*SD* = 12.53 years), with 49.8% of the sample being male (50.2% female), 22.9% Colombian nationals, and 77.1% Spanish nationals.

### Measures

The instrument considered, among other scales, an entrepreneurial orientation scale and a curiosity scale.

#### Entrepreneurial Orientation Scale

Adapted to Spanish by Boada-Grau et al. (2016) on the basis of its original version, developed by Lee et al. (2011), it is a Likert-type scale that, based on 12 items, measures the degree of agreement or disagreement on different levels (on a scale of 1–5, with 1 = completely disagree, and 5 = completely agree). These assess entrepreneurial orientation in four sub-dimensions: autonomy (related to self-sufficiency in facing challenges), innovation (related to enjoyment and orientation to work on new things), risk-taking (related to the willingness to face difficulties in the future), and aggressive competitiveness (related to perseverance and belief in success). Examples of sentences considered in the items are “I do not want to be financially supported by my parents, family, etc., because I am already an adult,” “I am more interested in starting my own business than in getting a job,” and “even if I start new businesses and fail many times, I will keep trying until I succeed.” According to Boada-Grau et al. (2016), the instrument had good psychometric properties, both in terms of validity and reliability. In this study, the overall reliability of the instrument, as shown by its Cronbach’s Alpha, was 0.74. The sum of the scores obtained in each of the 12 items that make up the scale was used to calculate the total score of the scale.

#### Curiosity Scale

Developed by Litman (2008), this scale integrates two scales that measure two different types of curiosity: the epistemic curiosity scale developed by Litman and Spielberger (2003) and the Feeling-of-Deprivation Scale, developed by Litman and Jimerson (2004). The first is related to curiosity linked to interest (I-type) and is associated with the anticipated pleasure felt when making new discoveries or acquiring new knowledge, simply for the intrinsic satisfaction of gaining it. The second refers to curiosity in terms of deprivation (D-type), and is associated with the need to acquire knowledge in order to reduce uncertainty and eliminate unwanted ignorance, which is why the accuracy and relevance of the information to be acquired are relevant in this case. The instrument has 10 items, five for the I-type curiosity and five for the D-type curiosity. The items correspond to sentences representing experiences to be rated according to the frequency with which they are experienced on a Likert-type scale from 1 to 4 (1 = almost never; 2 = sometimes; 3 = often; and 4 = almost always). These sentences are for instance “I enjoy learning unfamiliar topics” and “I get frustrated when I cannot solve a problem, so I try harder.” Litman (2008) stated that the instrument had good psychometric properties, both in terms of validity and reliability. In this study, the overall reliability of the instrument, as shown by its Cronbach’s Alpha, was 0.74 for the I-type curiosity and 0.71 for the D-type curiosity. The total score for each dimension of the scale was calculated based on the sum of the scores obtained in each of the five items that make up the scale.

The participants’ perceived likelihood of starting their own business in the next 5 years was also measured using the statement “estimate the probability of starting your own business in the next 5 years” (PSB), which could be rated on a scale from 0 to 10, where 0 corresponded to “not at all,” 5 to “neutral,” and 10 to “very likely.” In terms of socio-demographic variables, participants were asked their age (in years) and gender (male/female). To assess the culture of the participants (Latin American or European), the country where the instrument was applied (Colombia/Spain) was taken into account.

### Data Analysis

First, descriptive analyses were carried out for all the variables involved in the study (calculation of minimum, maximum, mean, and SDs). Also, a bivariate correlation analysis (Spearman’s rho) was then performed among all the variables, in order to observe how they were associated. Next, two simple moderation analyses were carried out to test the moderating role of both types of curiosity in the relationship between EO and PSB. Then, a double moderation analysis was carried out in order to determine which sociodemographic variables (age, gender, and culture) moderates the moderating effect of curiosity in the relationship between entrepreneurial orientation and the perceived possibility of setting up a business. The statistical analyses were carried out using the IBM-SPSS v.24 program and the PROCESS for SPSS v2.10 modelling tool (Hayes, 2018).

## Results

### Descriptive Results

Table 1 shows the minimum and maximum scores, the mean, and the SD of each of the variables considered in the study.

**Table 1:** Descriptive analysis of the study variables.

	Min.	Max.	*M*	*SD*
1. EO	12.00	60.00	38.68	7.69
2. I-type curiosity	5.00	20.00	15.61	3.13
3. D-type curiosity	5.00	20.00	15.04	3.07
4. PSB	0.00	10.00	4.72	2.56

Table 2 presents the Spearman correlation coefficients (Spearman rho), among all the variables. As it can be observed, all associations were positive and statistically significant (value of *p* < 0.01), and the I-type curiosity showed a stronger association than the D-type, with both EO and PSB.

**Table 2:** Bivariate correlation matrix (Spearman’s rho) for the study variables.

	1.	2.	3.	4.
1. EO	1			
2. I-type curiosity	0.41^*^	1		
3. D-type curiosity	0.28^*^	0.52*	1	
4. PSB	0.53^*^	0.31^*^	0.15^*^	1

* The correlation is significant at the 0.01 level (bilateral).

### Simple Moderation Analysis

This section presents the results of the simple moderation analyses performed. These models considered PSB as a dependent variable, EO as an independent variable and the I-type curiosity and D-type curiosity as moderating variables. A BCa bootstrapped CI based on 5,000 samples was used to calculate the CIs of all the models used. The mean, low, and high values of the moderating variables considered their mean plus/minus a SD.

#### I-Type Curiosity as a Moderator of the Relationship Between Entrepreneurial Orientation and Perceived Probability of Starting a Business

Table 3 shows the results of the linear regression model that considers PSB as a dependent variable, and EO, I-type curiosity, and the interaction between them, as independent variables.

**Table 3:** Linear model of predictors of Probability of Starting a Business (PSB), considering perceived I-type curiosity (*R*^2^ = 27.41%, *p* < 0.001).

	*B*	95% CI	*SE B*	*t*	*p*
Constant	−0.37	[−3.00, 2.26]	1.34	−0.27	0.79
EO	0.13	[0.06, 0.20]	0.04	3.69	<0.001
I-type curiosity	0.09	[−0.09, 0.26]	0.09	0.95	0.34
EO × I-type curiosity	0.00	[0.00, 0.00]	0.00	0.21	0.83

In this case, the interaction was not statistically significant (value of *p* = 0.83). That is, there is no evidence to argue that the I-type curiosity moderates the relationship between EO and PSB.

#### D-Type Curiosity as a Moderator of the Relationship Between Entrepreneurial Orientation and Perceived Probability of Starting a Business

Table 4 shows the results of the linear regression model that considers PSB as a dependent variable, and EO, I-type curiosity, and the interaction between them, as independent variables.

**Table 4:** Linear model of predictors of PSB, considering perceived D-type curiosity ( $R^{2}$ = 26.415%, *p* < 0.001).

	*B*	95% CI	*SE B*	*t*	*p*
Constant	3.01	[0.23, 5.77]	1.41	2.13	<0.05
EO	0.08	[0.01, 0.15]	0.04	2.25	<0.05
D-type curiosity	−0.19	[−0.37, 0.00]	0.09	−1.99	<0. 05
EO × D-type curiosity	0.01	[0.00, 0.01]	0.00	2.12	<0.05

**Figure 1:**
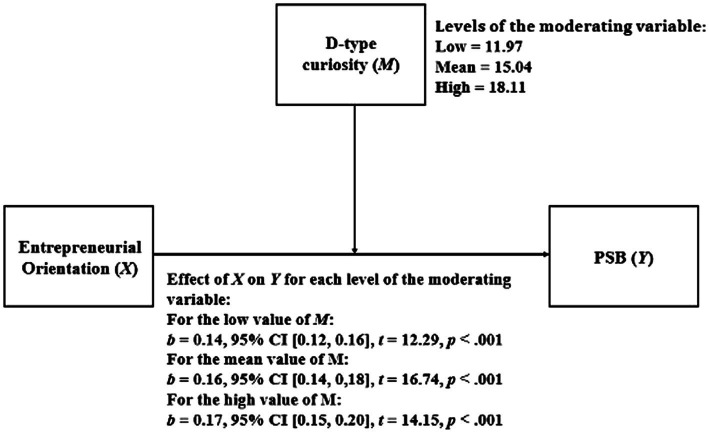
Simple moderation model considering D-type curiosity as moderator.

The fact that the interaction between independent variables was statistically significant for the model (value of *p* < 0.05) means that the moderation is statistically significant. An analysis was then carried out how the relationship between EO and PSB varied for the different levels of D-type curiosity. The results of this analysis are presented in Figure 1. As shown, as the D-type curiosity increases, the relationship between EO and PSB becomes stronger.

### Double Moderation Analysis

**Figure 2:**
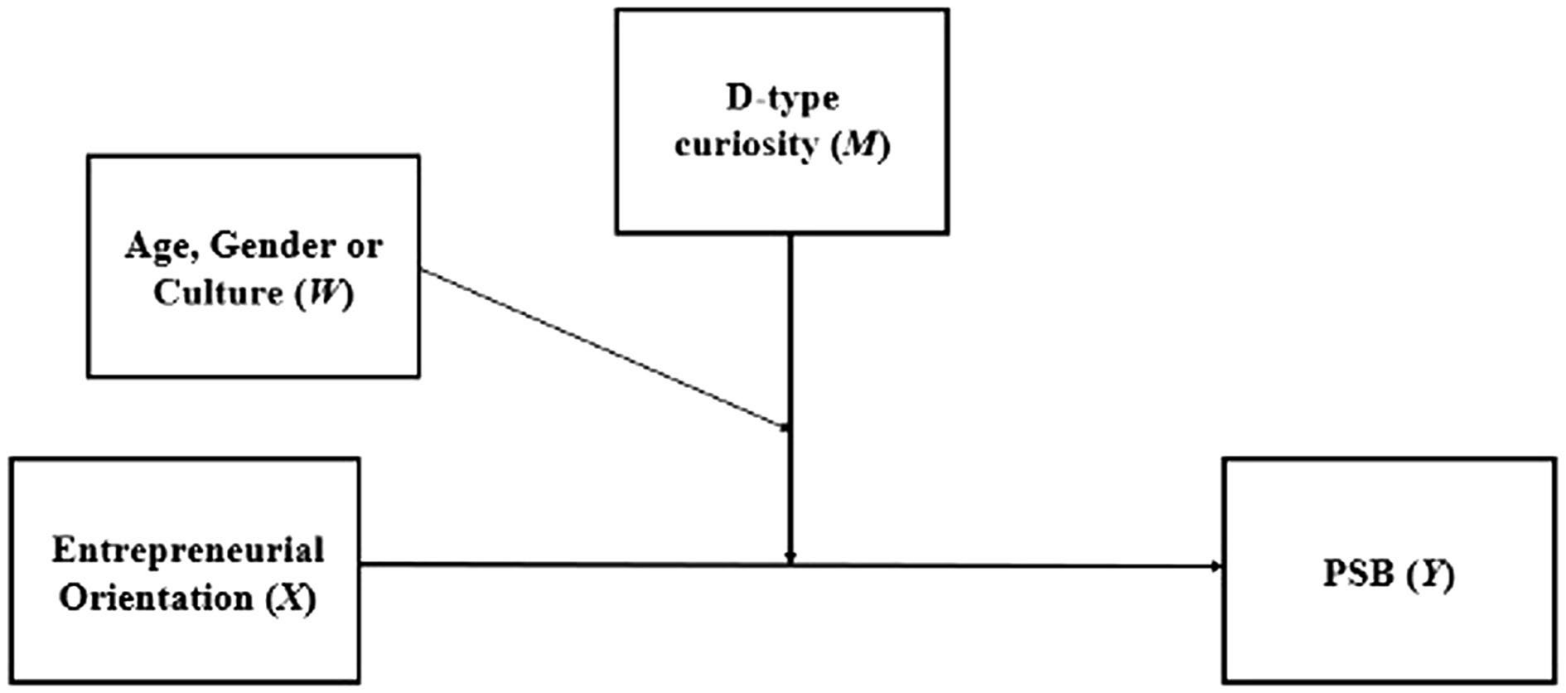
Conceptual model of double moderation analysis.

The results of the double moderation analyses are presented below. They were performed to observe how age, gender, and culture, can moderate the moderation of D-type curiosity in the relationship between EO and PSB (see Figure 2).

**Figure 3:**
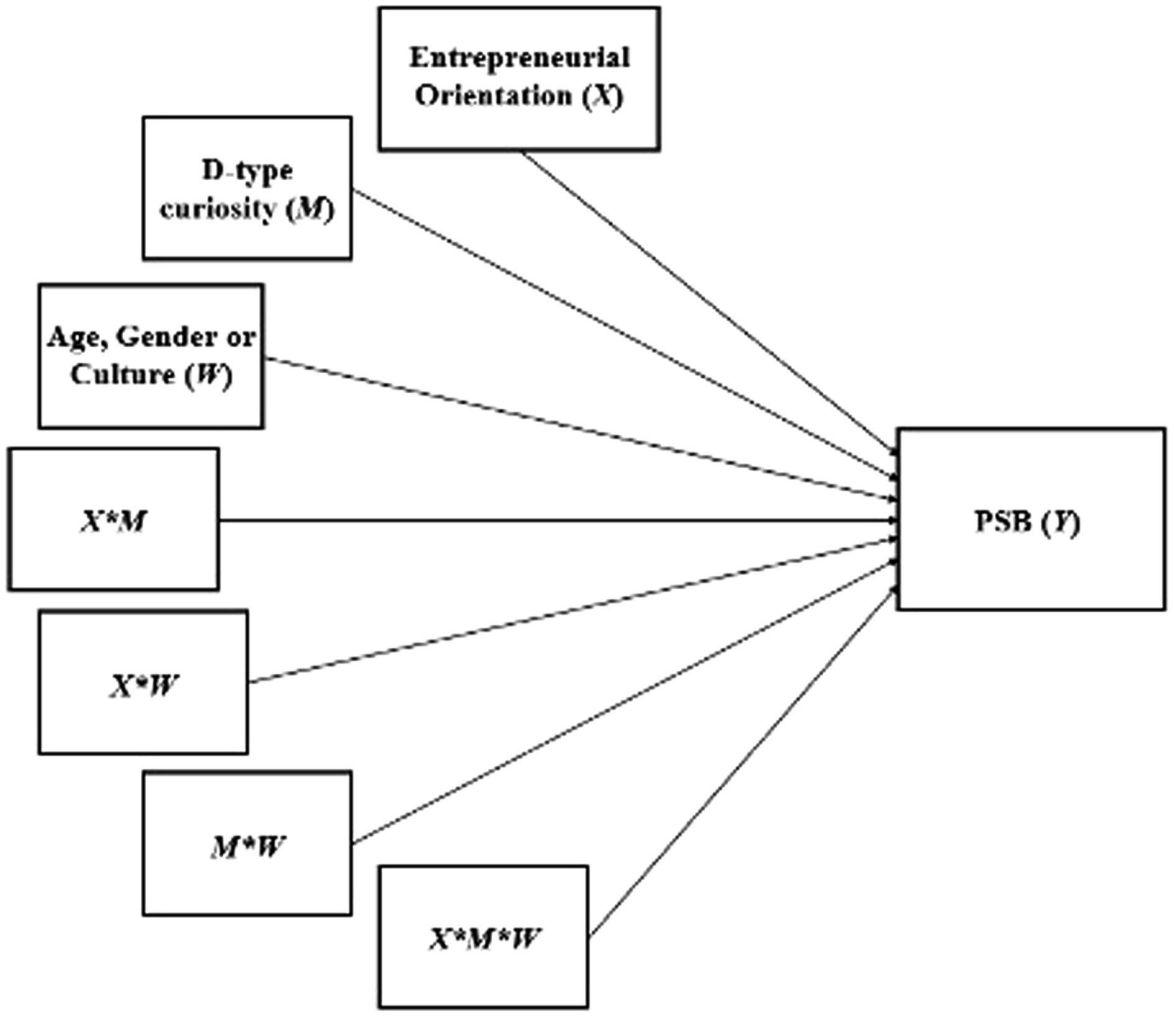
Multiple linear regression analysis for the double moderation model.

This procedure considers a multiple linear regression analysis, which includes as a dependent variable the PSB, and as independent variables the EO, the D-type curiosity, one of the sociodemographic factors (age, gender, or culture), and all possible combinations between these three variables (including the triple combination), as presented in Figure 3.

As in the previous analyses, a BCa bootstrapped CI based on 5,000 samples was used to calculate the CIs of all the models used. Due to the quantity and complexity of the models, only the results of the triple interaction between the EO, the D-curiosity, and each of the sociodemographic factors are presented, as this is the only factor that can show if the double moderation is statistically significant. These results are presented in Table 5.

**Table 5:** Statistical significance of the interaction between Entrepreneurial Orientation (EO), D-type curiosity, and the sociodemographic factors.

	*b*	95% CI	*SE B*	*t*	*p*
EO × D-type curiosity × Age	0.00	[0.00, 0.00]	0.00	2.69	<0.01
EO × D-type curiosity × Gender	0.00	[−0.01, 0.01]	0.01	0.93	0.35
EO × D-type curiosity × Culture	0.00	[−0.01, 0.01]	0.00	0.91	0.36

Regarding the sociodemographic variables, neither culture nor gender showed a moderating effect on the moderation exerted by the D-type curiosity in the relationship between EO and PSB. The double moderations of gender and culture were not statistically significant (value of *p* = 0.35 and 0.36, respectively). Table 6 shows how the variables interacted in the model that was statistically significant. The mean, low, and high values of the moderating variables considered their mean plus/minus a standard deviation.

**Table 6:** Conditional effect of EO on PSB at different levels of D-type curiosity and age.

Age	D-type curiosity	*b*	*SE B*	*t*	*p*	*LLCI*	*ULCI*
26.35	11.96	0.11	0.01	7.71	<0.001	0.08	0.14
26.35	15.04	0.11	0.01	7.63	<0.001	0.08	0.14
26.35	18.11	0.11	0.02	5.85	<0.001	0.07	0.14
38.88	11.96	0.13	0.01	10.42	<0.001	0.11	0.16
38.88	15.04	0.15	0.01	15.45	<0.001	0.13	0.17
38.88	18.11	0.17	0.01	13.20	<0.001	0.14	0.19
51.41	11.96	0.16	0.02	7.80	<0.001	0.12	0.20
51.41	15.04	0.19	0.01	13.68	<0.001	0.16	0.22
51.41	18.11	0.23	0.02	12.81	<0.001	0.19	0.26

As shown, for the younger segment, the different levels of D-type curiosity do not modify the strength of the relationship between EO and PSB, while for the middle-aged and older segments (especially for the latter), as the levels of D-type curiosity increase, so does the strength of the relationship between EO and PSB (especially in the older segment).

## Discussion and Conclusion

Considering that there was not much information regarding the moderating role of curiosity in the relationship between entrepreneurial orientation and actual actions related to entrepreneurship (such as starting a business), the aim of this study was to analyze this possible moderating role for different types of curiosity: I-type curiosity (linked to the intrinsic satisfaction of obtaining knowledge) and D-type curiosity (linked to the need of knowing for the purpose of solving a problem).

### I-Type and D-Type Curiosity as a Simple Moderator of the Relationship Between EO and PSB

This study showed that only the D-type curiosity moderated the relation between the EO and the PSB. The higher the levels of D-type curiosity, the stronger the relation between the EO and PSB. This makes sense as, considering the D-type curiosity corresponds to a need to acquire knowledge for a problem-solving purpose, it can be said that this type of need is goal-orientated (Litman, 2019). Thus, people who search relevant and pertinent knowledge in order to solve concrete problems, would show higher probabilities of setting up a business (in practical terms) using their entrepreneurial orientation. In contrast, the I-type curiosity component did not show a moderating effect on the relationship under study. This could be explained by the fact that this type of curiosity is associated with the need to acquire knowledge simply for the intrinsic satisfaction of obtaining it and not in terms of achieving a goal, hence, with a contemplative rather than transformative vision of reality (Litman, 2019). It would therefore be logical to think that people with higher levels of this type of curiosity focus more on understanding how the world works rather than doing something about it. This finding could have particular relevance in entrepreneurship education and training. In this way, it should be considered to strengthen psychological aspects that are linked to the achievement of specific goals, before strengthening psychological aspects linked to obtaining knowledge that only generates satisfaction intrinsically.

### I-Type and D-Type Curiosity and Their Interaction With Sociodemographic Variables, as Moderators of the Relationship Between EO and PSB

In addition, the aim was also to study how socio-demographic factors, such as age, gender, and culture, might moderate the moderation in question. This was only analyzed for the D-type curiosity, as it was the only one that showed a statistically significant moderation of the relationship in question. Results showed that age was the only factor with a moderating effect on the moderation under study. This suggests, firstly, that the effects of the D-type curiosity in entrepreneurship may be cross-cutting across gender and culture. In terms of age, for the younger segment, the different levels of D-type curiosity do not modify the strength of the relationship between EO and PSB, while for the middle-aged and older segments (especially for the latter), as the levels of D-type curiosity increase, so does the strength of the relationship between EO and PSB (especially in the older segment). This could be explained by arguing that the curiosity of younger people could be linked more to their enthusiasm than their problem-solving attitude, given that during this life stage they are more open to new experiences (Schwaba et al., 2018) and, generally, to higher levels of divergent thinking (Fusi et al., 2021). In terms of older people, the opposite could be true, as curiosity and EO could be linked to more convergent thinking and more focused practical behavior. However, beyond attitude factors, the moderation due to age on this matter could be explained on the basis of more tangible elements. Generally speaking, young people tend to have less stability and financial freedom. As a result, apart from their convictions, they might encounter more barriers when trying to set up a business, even when they focus on finding knowledge directly linked to the resolution of concrete problems (D-type curiosity). This finding could also have particular relevance in education and training in entrepreneurship, since it shows that the psychological skills to intervene to improve success in the field of entrepreneurship, affect the results of individuals in different ways, depending on their age.

## Conclusion

The results of this study can be framed within the field of entrepreneurial psychological capital. Psychological capital is defined as a positive psychological state that is mainly based on four elements: self-efficacy, optimism, hope, and resilience (Newman et al., 2014). If psychological capital is applied to the field of entrepreneurship, it is possible to see how certain psychological characteristics can be part of the psychological resources people have in order to successfully face the challenges of entrepreneurship. Some studies show that psychological capital is positively associated with entrepreneurs’ business success (Baluku et al., 2016; Wang et al., 2018). Baron et al. (2016) also demonstrated that high levels of psychological capital lead to low levels of stress in entrepreneurs, while Wang et al. (2018) showed how psychological capital is associated to entrepreneurs’ job satisfaction. In turn, curiosity (in particular D-type curiosity), can be associated with elements of psychological capital such as self-efficacy (with orientation as a common goal) and resilience (with orientation to persevere when facing difficulties as a common goal). In this context, the D-type curiosity can be associated with entrepreneurial psychological capital and, therefore, with higher levels of entrepreneurial orientation and probability of starting a business, as shown by the results of this study.

This study has some limitations. One of them is its cross-cutting design. If a longitudinal design were available, it would be possible to better study how variables are related in terms of sequentiality, i.e., which variables precede which. Moreover, an experimental design would make it possible to analyze the relationship between the study variables in terms of causality. Also, actually measuring people’s entrepreneurial actions would be more objective than asking them about their perception of future actions (such as their perceived probability of starting a business). In this way, measurements would not be affected by people’s biases or expectations (positive or negative) about their own performance. Future studies should consider these limitations to obtain a more precise understanding of the entrepreneurship phenomenon.

## Data Availability Statement

The raw data supporting the conclusions of this article will be made available by the authors, without undue reservation.

## Ethics Statement

Ethical review and approval was not required for the study on human participants in accordance with the local legislation and institutional requirements. The patients/participants provided their written informed consent to participate in this study.

## Author Contributions

NB and AR: preparation of the theoretical framework, methodological framework, and data analysis. LA-C, MB-C, and MV-V: data collection, database construction, discussion elaboration, and general revision of the manuscript. All authors contributed to the article and approved the submitted version.

## Conflict of Interest

The authors declare that the research was conducted in the absence of any commercial or financial relationships that could be construed as a potential conflict of interest.

## Publisher’s Note

All claims expressed in this article are solely those of the authors and do not necessarily represent those of their affiliated organizations, or those of the publisher, the editors and the reviewers. Any product that may be evaluated in this article, or claim that may be made by its manufacturer, is not guaranteed or endorsed by the publisher.
